# Recombinant Pseudorabies Virus (PRV) Expressing Firefly Luciferase Effectively Screened for CRISPR/Cas9 Single Guide RNAs and Antiviral Compounds

**DOI:** 10.3390/v8040090

**Published:** 2016-03-29

**Authors:** Yan-Dong Tang, Ji-Ting Liu, Qiong-Qiong Fang, Tong-Yun Wang, Ming-Xia Sun, Tong-Qing An, Zhi-Jun Tian, Xue-Hui Cai

**Affiliations:** 1 The Key Laboratory of Veterinary Public Health, Ministry of Agriculture, State Key Laboratory of Veterinary Biotechnology, Harbin Veterinary Research Institute of the Chinese Academy of Agricultural Sciences, Harbin 150001, China; tangyandong2008@163.com (Y.-D.T.); liujiting1989@163.com (J.-T.L.); fang19922020@163.com (Q.-Q.F.); sdndwty@163.com (T.-Y.W.); qzsmx122@163.com (M.-X.S.); antongqing@hvri.ac.cn (T.-Q.A.); tzj@hvri.ac.cn (Z.-J.T.); 2 College of Animal Science and Technology, Jilin Agriculture University, Changchun 130018, China

**Keywords:** pseudorabies virus, firefly luciferase, CRISPR/Cas9, chloroquine, Cyclosporine A

## Abstract

A Pseudorabies virus (PRV) variant has emerged in China since 2011 that is not protected by commercial vaccines, and has not been well studied. The PRV genome is large and difficult to manipulate, but it is feasible to use clustered, regularly interspaced short palindromic repeats (CRISPR)/Cas9 technology. However, identification of single guide RNA (sgRNA) through screening is critical to the CRISPR/Cas9 system, and is traditionally time and labor intensive, and not suitable for rapid and high throughput screening of effective PRV sgRNAs. In this study, we developed a recombinant PRV strain expressing firefly luciferase and enhanced green fluorescent protein (EGFP) as a reporter virus for PRV-specific sgRNA screens and rapid evaluation of antiviral compounds. Luciferase activity was apparent as soon as 4 h after infection and was stably expressed through 10 passages. In a proof of the principle screen, we were able to identify several PRV specific sgRNAs and confirmed that they inhibited PRV replication using traditional methods. Using the reporter virus, we also identified PRV variants lacking US3, US2, and US9 gene function, and showed anti-PRV activity for chloroquine. Our results suggest that the reporter PRV strain will be a useful tool for basic virology studies, and for developing PRV control and prevention measures.

## 1. Introduction

Pseudorabies virus (PRV) belongs to the Herpesviridae family [1,2] and is the etiological agent of pseudorabies (PR), also known as Aujeszky’s disease. PR causes substantial economic losses to the global swine industry [1], but has been largely controlled for at least 30 years using the Bartha-K61 vaccine. However, a novel PRV variant has emerged in China, and the Bartha-K61 vaccine has failed to provide complete protection [3,4,5,6]. Full-length genomic sequencing demonstrated that the PRV variant causing the outbreak belonged to a novel genotype [7]. Given the urgency of the outbreak and the need for a new vaccine, additional studies of PRV variants are critical. 

Due to its large genome, PRV has been manipulated for basic virology studies using homologous recombination (HR) [8] or bacterial artificial chromosome (BAC) techniques [9]. Both traditional methods have considerable drawbacks. For example, the frequency and efficacy of the expected recombination by HR is quite low; and BAC mutagenesis is only available for virus isolates for which a useful BAC has been produced. Therefore, CRISPR/Cas9 can be applied to new isolates as they emerge in nature, with the only delay being the need to have sequence data for design of guide RNAs. In addition, inserting drug selection markers or parts of BAC plasmids into the viral genome may affect viral function [10]. However, genetic manipulation is essential for identifying gene function and for vaccine development.

With the development of alternative technologies such as zinc-finger nucleases (ZFNs), transcription activator-like effector nucleases (TALENs) and CRISPR/Cas9 [11,12], genome editing has become significantly less complicated. These approaches use a nuclease to specifically target a gene, cleaving the DNA to induce double-stranded breaks at the target site. The DNA break triggers cellular DNA repair mechanisms, including error-prone non-homologous end joining (NHEJ) and homology-directed repair (HDR) [13]. Customizing gene disruption using either ZFNs or TALENs requires the design of specific proteins to target each dsDNA site [14,15], which requires several weeks and is labor and time intensive. However, gene knock out or gene knock in recombinants can be obtained within a short period by simply transfecting the CRISPR/Cas system, which is both efficient and convenient [16,17,18]. 

The CRISPR/Cas9 system—derived from the bacterial adaptive immune system—has been used to successfully edit many viruses [18,19,20,21]. One key factor affecting DNA editing using the CRISPR/Cas9 system is effective screening for single guide RNAs (sgRNAs), which has to be validated by amplified fragment length polymorphism (AFLP), T7 endonuclease I assay (T7E1), surveyor mismatch cleavage assays, or DNA sequencing. These methods are time consuming, laborious, and minimally sensitive [22]. The PRV genome contains a high GC content, which is challenging for PCR amplification. Therefore, all of the traditional approaches are suboptimal for effectively screening for PRV sgRNAs.

Here, to address the shortcomings inherent in screening for PRV sgRNAs, we have taken a novel approach and use a PRV containing firefly luciferase to validate PRV-specific sgRNA screening. We then applied the reporter virus to develop novel inactivated PRV strains and to demonstrate its utility in antiviral screening assays.

## 2. Materials and Methods

### 2.1. Cell Lines and Viruses

Vero cells and MARC 145 cells were cultured in Dulbecco’s modified Eagle’s medium (DMEM; GIBCO, Grand Island, NY, USA). All culture media was supplemented with 10% heat-inactivated fetal bovine serum (FBS, GIBCO, Life technologies) and antibiotics (0.1 mg/mL streptomycin and 100 IU/mL penicillin). The PRV HeN1 strain (GenBank accession number: KP098534.1) was described previously [5,7]. PRRSV HuN4 strain (GenBank accession number: EF635006.1). 

### 2.2. Generation of the HR Plasmid and CRISPR/Cas9 sgRNA Plasmids

The HR plasmid was constructed in the pcDNA3.1 (+) expression vector (Clontech, PaloAlto, CA, USA). First, pcDNA3.1 (+) was digested using *PmeI* to remove the multiple cloning site (MCS) between it (from *Afl II* to *Apa I*). Then, site mutagenesis was utilized to insert the 5′ *BamHI* and 3′ *NotI* sites that flank the neomycin resistance gene. In addition, sites for *EcoRI*, *SalI*, and *XhoI* were created followed by an SV40 poly(A) signal sequence. Next, *BamHI* and *NotI* were used to remove the *EGFP* gene from the pEGFP-N1 plasmid (Clontech, PaloAlto, CA, USA) and used to replace the neomycin gene (Tang-EGFP vector). The firefly luciferase gene was then amplified from the pGL3-Basic vector (Promega, Madison, WI, USA) and cloned into the Tang-EGFP vector between the *NheI* and *PmeI* sites. The right and left homologous arms were cloned from the PRV HeN1 strain by PCR and inserted between the CMV promoter and SV40 poly (A) signal. The final plasmid was termed the Tang-Luc-EGFP-HR vector. sgRNAs involved in this study were designed using the online CRISPR Design Tool [23], and target the *gE*, *US2*, *US3* and *US9* genes open reading frames. A human codon-optimized SpCas9 and the chimeric guide RNA expression plasmid PX330 were gifts from Feng Zhang [17,24]. The PX330 plasmid was digested using BbsI (Thermo scientific fermentas, Waltham, MA, USA) and the CRISPR/Cas9 constructs were constructed. All of the constructs in this study were verified by sequencing. The primers used are provided in Table 1.

### 2.3. Recombination and Purification of the Luciferase Tagged PRV

The PRV HeN1 genome was extracted as previously described [7,25]. Vero cells were co-transfected with 1μg of the PRV genome, 1 μg of cas9 plasmid gRNA-gE1, and 3 μg of the Tang-Luc-EGFP-HR plasmid using the X-tremeGENE HP DNA transfection reagent (Roche, Basel, Switzerland) according to the manufacturer’s instructions. Forty-eight hours post transfection, the cells were collected and then subjected to three freeze-thaw cycles. Recombinant PRV was purified from the cell lysates by plaque purification (EGFP + selected) in Vero cells overlaid with 1% low-melting point agarose and 2% FBS in DMEM. After 10 rounds of purification, all of the plaques were EGFP+ upon examination by fluorescent microscopy.

### 2.4. In Vitro Growth Properties

Viral titers were determined by plaque forming unit (PFU) or the 50% tissue culture infection dose (TCID_50_) in Vero cells. To compare the growth kinetics of the parental and EGFP expressing viruses, Vero cells were infected with wild type PRV HeN1 or recombinant PRV-Luc-EGFP at a dose of 200 TCID_50_. The cells were collected at 24, 48, 72, and 96 h post infection (hpi), and the viral titer was determined by PFU or TCID_50_ at the time points indicated.

### 2.5. Transfection and Western Blot

Cells were transiently transfected with the indicated plasmids using the X tremeGENE HP DNA transfection reagent (Roche, Basel, Switzerland) according to the manufacturer’s instructions. Forty-eight (48) hpi, the cells were collected and washed once with PBS, and then lysed in RIPA Lysis Buffer containing a protease inhibitor cocktail (Roche, Basel, Switzerland). The proteins in the cell lysates were separated by SDS-PAGE, transferred to PVDF membranes (Millipore, Milford, MA, USA), and probed with the indicated antibodies for detection. 

### 2.6. CRISPR/Cas9 sgRNA Screening and Antiviral Assay

The CRISPR/Cas9 plasmids were transfected into Vero cells, and 12 h later the cells were infected with 0.01 multiple of infection(MOI) PRV HeN1 or PRV-Luc-EGFP. The expression levels of the indicated proteins were assessed at the indicated time points post infection by Western blot, or luciferase activity was measured using the luciferase assay system (Promega, Madison, WI, USA). For the antiviral assay, monolayers of 90% confluent Vero cells in 12-well plates were treated with Cyclosporine A (CsA) (Beyotime, Jiangsu, China) or chloroquine (Sigma, St. Louis, MO, USA) for 4 h and then infected with PRV HeN1 or PRV Luc-EGFP. The effects of the antiviral compounds were determined based on viral protein expression and luciferase activity as described above. For CsA inhibit porcine reproductive and respiratory syndrome virus (PRRSV) assays, MARC 145 cells were infected with 0.1 MOI PRRSV HuN4 strain and incubated 12 h at 37 °C. Cells were fixed with 80% ethanol for 30 min and stained with SR-30 FITC conjugated mAb (Rural Technologies, Brookings, SD, USA) for 3 h. and images were acquired with a fluorescent microscope. The effect of CsA and chloroquine on cell viability was determined by Trypan Blue staining (Sigma, St. Louis, MO, USA).

## 3. Results

### 3.1. PRV-Luc-EGFP was Successfully Constructed

**Figure 1:**
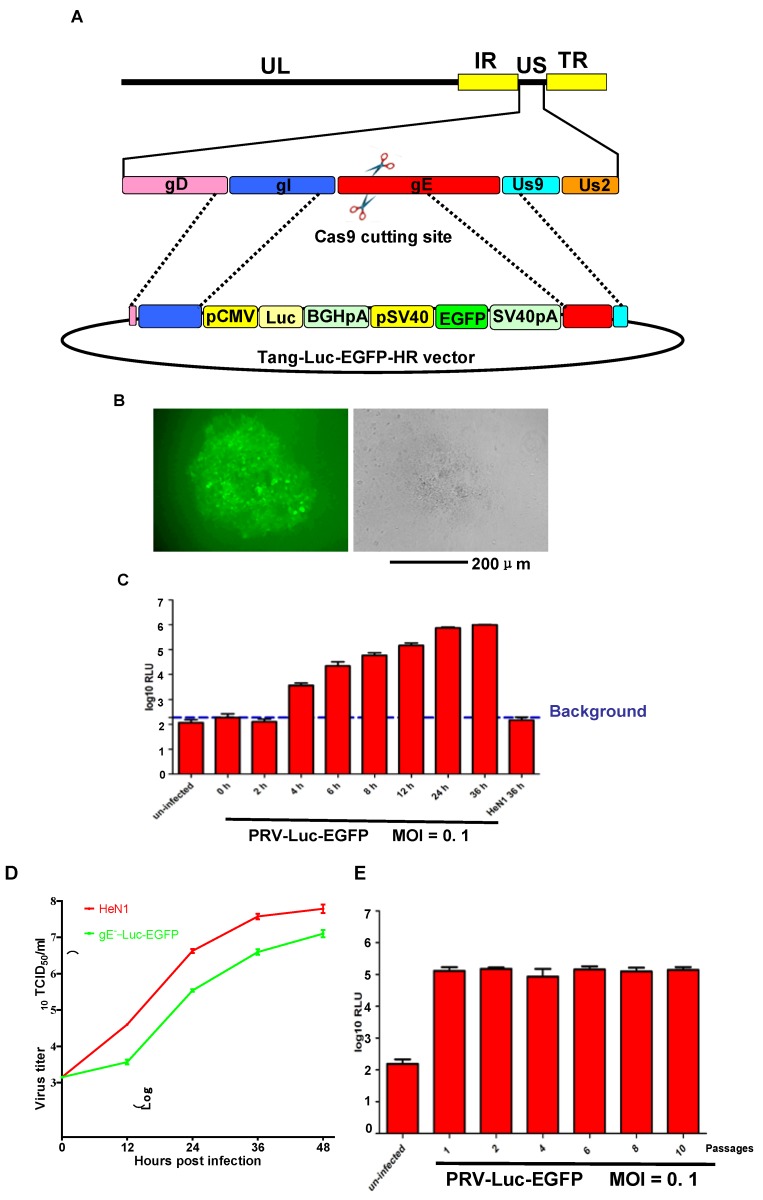
Construction and identification of PRV-Luc-EGFP. (**A**) Schematic showing the recombination sites used to modify the PRV genome and insert the Tang Luc-EGFP-HR donor vector using the CRISPR/Cas9 system; (**B**) Representative image showing the plaque assay for PRV-Luc-EGFP purification. The left panel shows fluorescent green plaque formation after seven rounds of plaque purification. The right panel shows the same area in bright field; (**C**) Luciferase expression in Vero cells at different time points post infection with PRV-Luc-EGFP (0.1 MOI); (**D**) One-step growth curve of the recombinant virus (PRV-Luc-EGFP; green) compared to the parental virus (HeN1; red); (**E**) Stability of PRV-Luc-EGFP. Luciferase activity was measured from passage 1 through passage 10.

As glycoprotein E (gE) is unnecessary for PRV replication *in vitro* [26,27], we chose this region to insert the firefly luciferase gene. First, plasmids were constructed containing both firefly luciferase and EGFP flanked by homologous arms designated Tang-Luc-EGFP-HR vector (Figure 1A). To generate the recombinant viruses, Vero cells were transfected with the purified PRV HeN1 genome, Tang-Luc-EGFP-HR, and gRNA-gE1 (a gE specific CRISPR/Cas9 construct that was used to improve the efficiency of the HR, our unpublished data). Forty-eight (48) hpi, the viruses were collected and plaque purified. After 10 rounds of purification, the expression of EGFP was confirmed by fluorescence microscopy (Figure 1B) and firefly luciferase expression was confirmed by luciferase activity (Figure 1C). The luciferase activity was detected as early as 4 hpi and reached a higher level at 36 h (Figure 1C). To determine whether the expression of firefly luciferase and EGFP influenced the replication of the PRV-Luc-EGFP virus, one-step growth curves were performed for PRV-HeN1 and PRV-Luc-EGFP. We found that PRV-Luc-EGFP replicated significantly slower than PRV-HeN1 (Figure 1D). We also evaluated the stability of the recombinant virus, and found that the luciferase activity from PRV-Luc-EGFP was stable through at least 10 passages (Figure 1E).

### 3.2. PRV-Luc-EGFP Was an Effective Tool for CRISPR/Cas9 sgRNA Screening

**Figure 2:**
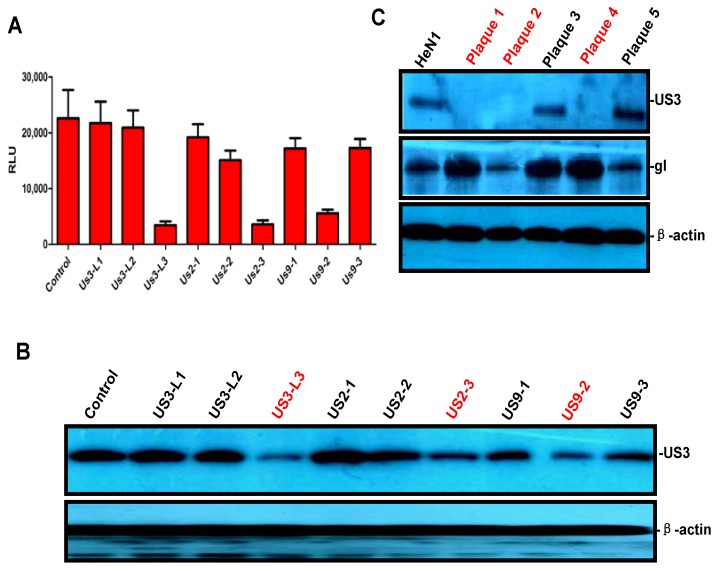

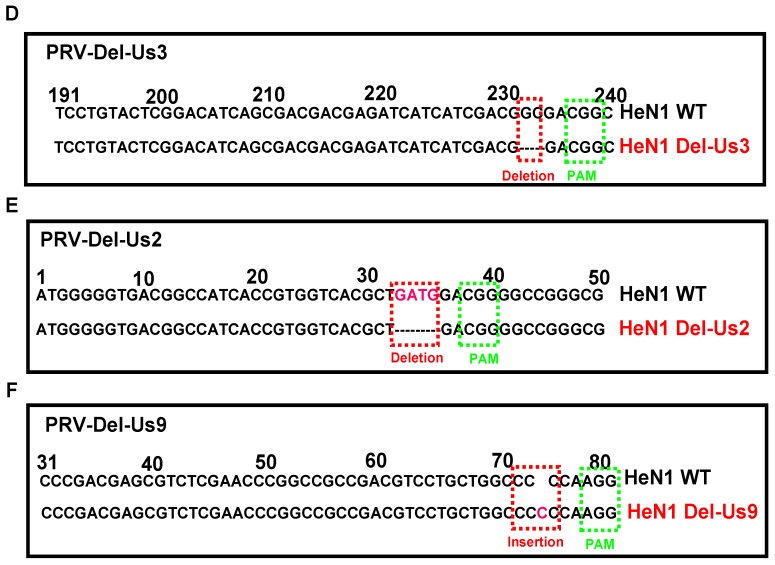
Effective sgRNA screening using PRV-Luc-EGFP and identification of PRV strains with the *US3*, *US2*, or *US9* genes inactivated. (**A**) sgRNA screening using PRV-Luc-EGFP. Cas9 constructs and a vector control were transfected individually into Vero cells. Twelve (12) h later, the cells were infected with PRV Luc-EGFP and 24 hpi luciferase activity was detected; (**B**) Representative image showing US3 expression by Western blot in PRV-HeN1 infected cells screening the same panel of sgRNAs as in (**A**); (**C**) Representative image showing US3 and gI expression in five randomly selected plaques that were treated with sgRNA US3-L3 by Western blot. Inactivated viruses were identified based on lack of US3 expression (e.g., plaque 4). Sequencing was used to confirm that the viruses considered inactivated had non-function genes for *US3* (**D**); *US2* (**E**); and *US9* (**F**). The images in A, B and C show representative results from three independent experiments.

To test whether PRV-Luc-EGFP could be used to screen for sgRNA, we designed and constructed several CRISPR/Cas9 constructs that were specific for *US3*, *US2*, and *US9*, respectively (Table 1). In short, effective CRISPR/Cas9 constructs would disrupt the PRV genome and inhibit viral replication, thereby decreasing luciferase activity. To test the designed sgRNAs, Vero cells were transfected with the CRISPR/Cas9 constructs, infected with PRV-Luc-EGFP 12 h later, and then luciferase activity was measured 24 hpi. As shown in Figure 2A, the sgRNAs US3-L3, US2-3, and US9-2 significantly reduced the amount of luciferase activity. To confirm the reduction in viral replication, we also assessed the effects of the sgRNAs on viral protein expression by Western blot. Consistent with the luciferase assay results, the cells transfected with the sgRNAs US3-L3, US2-3, and US9-2 had significantly decreased levels of US3 (Figure 2B). DNA breaks trigger the NHEJ repair pathway, which sometimes result in the introduction of crippling mutations and then inactivates gene expression. We next tested whether the sgRNAs would inactivate specific *PRV* genes. PRV-HeN1 treated with US3-L1, US2-3, and US9-2 was collected for plaque purification. Five (5) purified plaques were randomly selected to determine whether they had been inactivated based on US3 expression, and of these 60% (3/5) had reduced or no US3 expression (Figure 2C). No disruption was observed in the gI proteins, indicating the target specificity of sgRNA (Figure 2C). One of the plaques lacking US3 expression (plaque 4) was sequenced and found to be lacking two base pairs in the *US3* region (Figure 2D). PRV strains lacking expression of US2 and US9 were generated using similar methods (Figure 2E,F). These results indicated that PRV-Luc-EGFP was a powerful tool for effectively screening for CRISPR/Cas9 sgRNA.

### 3.3. Evaluating the Antiviral Activity of Chloroquine and CsA

**Figure 3:**
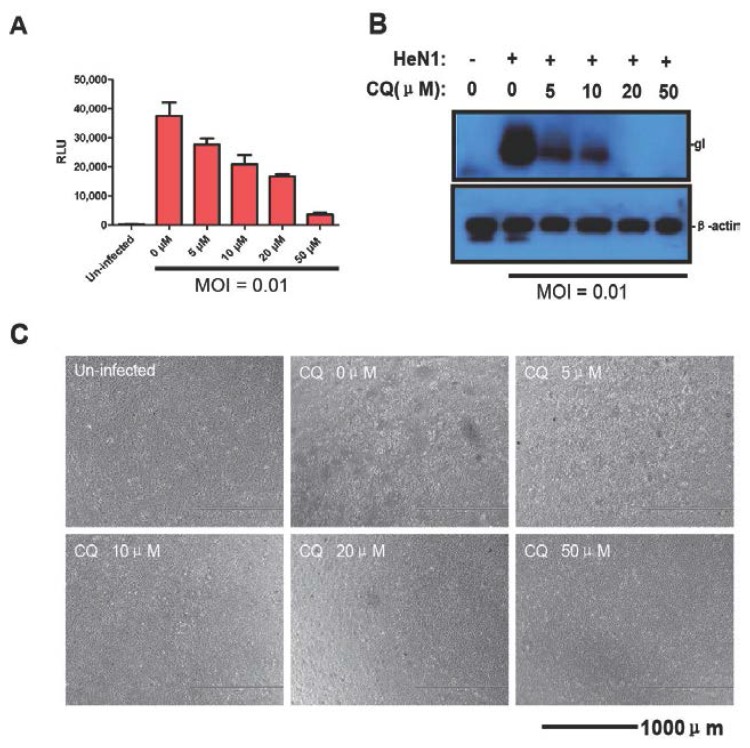
Chloroquine inhibits PRV replication. (**A**) Chloroquine inhibits PRV Luc-EGFP replication. Vero cells were pretreated with the indicated amounts of chloroquine and 4 h later infected with PRV-Luc-EGFP (0.01 MOI). After 24 h luciferase activity was evaluated; Chloroquine inhibits PRV HeN1, which is detected by gI expression (**B**); and CPE (**C**). The images show representative results from three independent experiments.

There are very few antiviral compounds that are effective against PRV *in vitro* and none *in vivo*. Thus, an effective *in vitro* assay for screening antiviral compounds against PRV would likely contribute to controlling PR. Therefore, as proof of principle, we evaluated the antiviral effects of chloroquine and CsA using PRV-Luc-EGFP and confirmed the results with PRV HeN1. Vero cells were pretreated for 4 h with chloroquine or CsA and then infected with PRV-Luc-EGFP for 24 h. The luciferase activity decreased in a dose dependent fashion with increasing chloroquine (Figure 3A–C).

**Figure 4:**
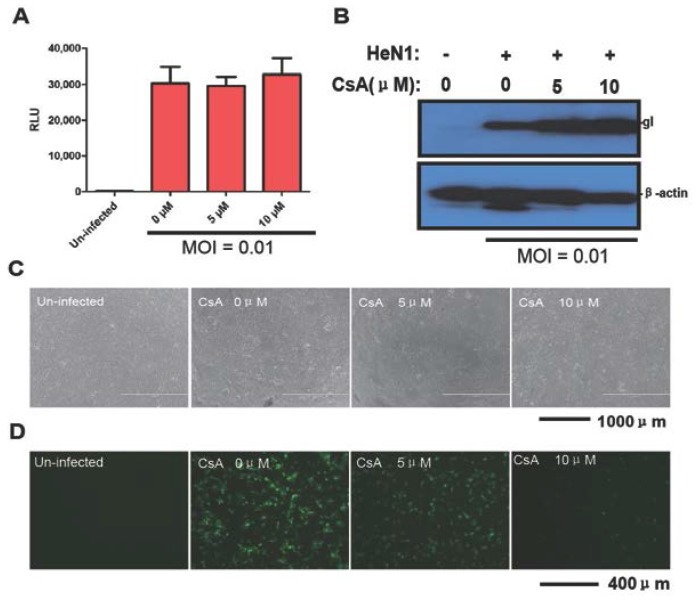
Cyclosporin A (CsA) has no effect on PRV replication. (**A**) Vero cells were pretreated with the indicated amounts of CsA and 4 h later were infected with PRV-Luc-EGFP (0.01MOI). After 24 h, luciferase activity was evaluated; (**B**) Vero cells were pretreated with the indicated amounts of CsA and 4 h later were infected with PRV HeN1 (0.01MOI). After 24 h, gI expression was evaluated by Western blot (representative image); (**C**) Representative images showing the cytopathic effect of HeN1 on Vero cells pretreated with different amount of CsA; (**D**) Marc145 cells were pretreated with the indicated amounts of CsA and then infected with 0.1 MOI PRRSV. Twelve (12) hpi, the N protein was detected by immunofluorescence. The images show representative results from three independent experiments.

In contrast, CsA did not have any inhibitory activity against PRV-Luc-EGFP (Figure 4A). Consistent with this, PRV HeN1 infection was not inhibited by CsA (Figure 4B,C). We simultaneously used PRRSV as a positive control for the antiviral effects of CsA. Using a FITC conjugated antibody directed against the N protein in PRRSV, CsA powerfully inhibited PRRSV replication (Figure 4D). Taken together, these results indicated that PRV-Luc-EGFP was a useful tool for screening antiviral compounds.

## 4. Discussion

Genetic manipulation of large, complex DNA viruses such as PRV is critical for understanding their biology and developing novel control strategies. Therefore, in this study, we developed a novel PRV strain expressing firefly luciferase for use as a tool in future studies. Luciferase expression was detectable as early as 4 hpi, suggesting that the reporter virus will be able to shorten assay time for antiviral screens and improve the assay sensitivity. In addition, when PRV genomes were cut by the CRISPR/Cas9 system, the luciferase expression was inhibited, indicating that the reporter virus is a powerful tool for identifying PRV specific sgRNAs, which will help us edit PRV genome more feasible. Finally, using the reporter virus, we successfully developed US2, US3, and US9 inactivated PRV strains, and demonstrated that chloroquine inhibits PRV replication, whereas CsA does not.

The CRISPR/Cas9 system has many distinct advantages as a genome editing tool including being both efficient and convenient compared to other methods [16,17,18]. We have previously utilized the DNA breaks caused by the CRISPR/Cas9 system that inhibit PRV replication to develop the gE^−^/gI^−^/TK^−^ PRV strain to screen for PRV sgRNA using plaque formation assays or Western blot; however, these methods require 3–4 days (our unpublished data revised in Virology). The current luciferase based reporter system yields results in a much shorter timeframe (4 h), allowing a more rapid pace of vaccine development and basic research. For large genome viruses, the CRISPR/Cas9 system was a usable tool to insert foreign reporter genes in the genome as well [18,21].

Chloroquine is a 9-aminoquinoline that is well-known for antimalarial activity and antiviral activity against some viral infections [28]. Exposure to a low intracellular pH is required for successful entry of many viruses. Chloroquine exerts direct antiviral effects by inhibiting pH-dependent steps of the virus lifecycle for some members of the flaviviruses, retroviruses, and coronaviruses [28]. Chloroquine has been showed to inhibit herpes simplex virus type 1 (HSV-1) replication [29], and may inhibit PRV may by same mechanisms, but this remains to be confirmed. CsA is a fungal metabolite that exerts profound effects on the immune system and is potentially a selective immuno-suppressive agent [30]. The antiviral effects of CsA originate from inhibiting virus replication by blocking cyclophilins, which are beneficial for some viruses [31,32]. CsA has been reported to inhibit replication of Human Immunodeficiency Virus-1 [33], Hepatitis C Virus [34,35], PRRSV, and Equine arteritis virus [31]. With regard to herpes viruses in particular, CsA has been shown to inhibit HSV-1 replication [36,37]. Interestingly, in our study, CsA did not show any inhibitory activity against PRV in Vero cells. The lack of antiviral CsA-mediated antiviral activity may be because PRV replication is not associated with cyclophilins in Vero cells.

In conclusion, we developed a luciferase tagged PRV, which was a powerful tool for screening for sgRNA and antiviral compounds. The reporter virus likely has utility for basic research into emerging PRV variants, and developing virus control and prevention measures.

## Figures and Tables

**Table 1:** Sequences of the Primers and sgRNAs Utilized in this Study.

Primers and sgRNAs	Sequences
CreBamHI	5′-CTTTTGCAAAAAGCTCCCGGGATCCTGTATATCCATTTTCG-3′
	5′-CGAAAATGGATATACAGGATCCCGGGAGCTTTTTGCAAAAG-3′
CreNotI	5′-GGATGATCCTCCAGCGGCCGCATCTCATGCTGGAG-3′
	5′-CTCCAGCATGAGATGCGGCCGCTGGAGGATCATCC-3′
CreEcoRI XhoI	5′-CTTATCATGTCTGAATTCCGTCGACCTCTAGCTCGAGCTTGG-3′
	5′-CAAGCTCGAGCTAGAGGTCGACGGAATTCAGACATGATAAG-3′
Luciferase	5′-CACGCTAGCCACCATGGAAGATGCCAAAAAC-3′
	5′-AGCTTTGTTTAAAC TTACACGGCGATCTTGCCGC-3′
HR L arm	5′-ACAAGATCTCCGGTCCGTAGCCTCCGCAGTA-3′
	5′-ACAACGCGTCGAAGCTCGGCCAACGTCATC-3′
HR R arm	5′-CCGGAATTCGGGCCGTGTTCTTTGTGGC-3′
	5′-CGGCTCGAGACTCGCTGGGCGTCTCGTTG-3′
sgRNA-gE1	5′-CACCGGGGCAGGAACGTCCAGATCC-3′
	5′-AAACGGATCTGGACGTTCCTGCCCC-3′
sgRNA-US3-1	5′-CACCGCCCCGACGAGATCCTGTACT-3′
	5′-AAACAGTACAGGATCTCGTCGGGGC-3′
sgRNA-US3-2	5′-CACCGGAGATCATCATCGACGGCGA-3′
	5′-AAACTCGCCGTCGATGATGATCTCC-3′
sgRNA-US3-3	5′-CACCGGAGATCATCATCGACGGCGA-3′
	5′-AAACTCGCCGTCGATGATGATCTCC-3′
sgRNA-US2-1	5′-CACCGACCGTGGTCACGCTGATGGA-3′
	5′-AAACTCCATCAGCGTGACCACGGTC-3′
sgRNA-US2-2	5′-CACCGGGGCGCATCCCCGCCTTCGT-3′
	5′-AAACACGAAGGCGGGGATGCGCCCC-3′
sgRNA-US2-3	5′-CACCGGGCGCACCCGGACCTGTGGA-3′
	5′-AAACTCCACAGGTCCGGGTGCGCCC-3′
sgRNA-US9-1	5′-CACCGCGACGTCCTGCTGGCCCCCA-3′
	5′-AAACTGGGGGCCAGCAGGACGTCGC-3′
sgRNA-US9-2	5′-CACCGGCCAGCAGGACGTCGGCGGC-3′
	5′-AAACGCCGCCGACGTCCTGCTGGCC-3′
sgRNA-US9-3	5′-CACCGGGGGTCCCTTGGGGGCCAGC-3′
	5′-AAACGCTGGCCCCCAAGGGACCCCC-3′
